# Cold-Pressed Insulation Boards from Recycled Cotton Fibers Using a Water-Borne PVAc–Starch Binder: Processing, Structure and Properties

**DOI:** 10.3390/ma19061097

**Published:** 2026-03-12

**Authors:** Tadeáš Zachara, Přemysl Šedivka, Vlastimil Borůvka, Kryštof Kubista, Tomáš Holeček, Martin Lexa, Lukáš Sahula, Adam Sikora

**Affiliations:** Faculty of Forestry and Wood Sciences, Czech University of Life Sciences Prague, Kamýcká 129, 165 00 Prague, Czech Republic; sedivka@fld.czu.cz (P.Š.); boruvkav@fld.czu.cz (V.B.); kkubista@fld.czu.cz (K.K.); holecekt@fld.czu.cz (T.H.); lexa@fld.czu.cz (M.L.); sahulal@fld.czu.cz (L.S.); sikoraa@fld.czu.cz (A.S.)

**Keywords:** fiber-based composites, recycled cotton fibers, cold pressing, water-borne binder, thermal parameters, moisture-related behavior, mechanical properties

## Abstract

This study investigates the valorization of post-consumer and post-industrial recycled cotton fibers from textile waste into porous fiber-based insulation composites using a low-temperature cold-pressing process and a water-borne hybrid binder based on polyvinyl acetate (PVAc) and modified cornstarch. Insulation boards were produced with target densities ranging from 300 to 340 kg·m^−3^, achieved by systematically adjusting the percentage weight fractions of recycled cotton fibers and binder components. The influence of board density on microstructure, inter-fiber bonding, and structure–property relationships was evaluated. The resulting boards exhibited thermal conductivity values between 0.0710 and 0.0739 W·m^−1^·K^−1^. Compressive strength measured at 10% relative deformation of the specimen thickness ranged from 46 to 162 kPa, while internal bond strength varied between 2 and 6 kPa. Water absorption decreased by approximately 18% with increasing density, indicating improved binder distribution and reduced open porosity. The PVAc–starch binder system enabled effective inter-fiber bonding without formaldehyde-based resins or energy-intensive curing, supporting a low-temperature and circular processing concept for textile waste valorization. Overall, the results demonstrate that recycled cotton fibers represent a viable feedstock for porous insulation composites combining balanced thermal, mechanical, and moisture-related performance with potentially reduced environmental impact.

## 1. Introduction

The built environment is responsible for approximately 37% of global annual CO_2_ emissions, with more than half of this originating from heat losses due to inadequately insulated building envelopes [1].

From a material design perspective, new sustainable materials should combine low thermal conductivity, mechanical integrity, and low-toxicity chemistry. In 2025, European Union legislation mandates the separate collection and recycling of textile waste under Directive 2008/98/EC, as amended by Directive 2018/851/EC. This regulatory framework demands the development of new functional materials for the cascading use of textile fibers into sustainable products with added value, for example new thermal insulation materials. Recycled lignocellulosic or textile fibers contribute to the circular economy by diverting post-consumer waste from landfills, while requiring minimal additional energy for processing [2].

Recycled cotton can be valorized through multiple pathways, including fiber-to-fiber routes, nonwoven products, or conversion into regenerated cellulose feedstocks. However, especially for post-consumer streams with heterogeneous composition (blends, finishes, and additives), high-purity recovery routes often require intensive sorting and additional chemical or separation steps, which can increase process complexity and energy demand [3]. In this context, building insulation represents a technically sound and scalable application that preserves the fibrous morphology and exploits the inherent structure–function advantages of cotton-based networks, namely high porosity and air retention, which are key drivers of low effective thermal conductivity in fibrous insulations [4]. Moreover, mechanical recycling routes are generally compatible with cotton and can retain sufficient fiber functionality for non-structural, high-volume products such as insulation, offering a pragmatic circular-economy option for textile fractions that are less suitable for high-grade textile reuse [5]. From an environmental perspective, the life-cycle literature also indicates that recycling-based textile management strategies can reduce impacts relative to virgin fiber production and disposal, supporting the rationale for cascading use of cotton waste into long-lived building products [6]. The existing literature has already explored several pathways for valorizing textile-waste fibers into functional composite panels, using both synthetic and more sustainable binder concepts. For example, recycled nonwoven wastes (cotton, polyester, and cotton/polyester blends) have been combined with epoxy matrices to produce panels targeting mechanical, thermal, and acoustic performance, demonstrating that textile-waste reinforcement can yield multifunctional composites but typically at the cost of petrochemical binder use [7]. More recently, waste cotton fabric has been processed into hot-pressed layered plates (waste-cotton-fabric reinforced polymer systems), where processing variables such as pressing temperature, waste-fabric fraction, and layer alignment were shown to strongly affect strength, water uptake, and thermal conductivity, indicating that both binder choice and architecture control are decisive for performance [8]. For insulation-relevant thermal behavior, post-consumer cotton in loose-fiber form has been systematically assessed across different bulk densities, underlining the sensitivity of effective thermal conductivity to consolidation level even when the feedstock remains nominally cotton [4].

Among such resources, post-consumer cotton exhibits an intrinsic solid-phase thermal conductivity of approximately 0.04 W·m^−1^·K^−1^ [4]. The primary challenge lies in transforming these hydrophilic, crimped fibers into cohesive, self-supporting boards without relying on formaldehyde-based or highly cross-linked synthetic resins.

Recent epoxy/flax-hemp laminates filled with waste glass dust achieved an effective thermal conductivity λ of approximately 0.30 W·m^−1^·K^−1^, but required a virgin epoxy matrix and curing at 120 °C [9]. Cellulose nanofiber aerogels crosslinked with melamine-urea-formaldehyde exhibited a λ of less than 0.025 W·m^−1^·K^−1^, though they were brittle and released free formaldehyde [10]. Cotton filter waste mats, hot-pressed with thermoplastic corn starch, reduced λ to 0.046 W·m^−1^·K^−1^, but their compressive strength remained under 20 kPa [11]. Epoxy syntactic foams containing hollow glass microspheres demonstrated hydrostatic pressure resistance and a λ of approximately 0.10 W·m^−1^·K^−1^, although at densities exceeding 500 kg·m^−3^ [12]. Hollow glass bead-filled carbon fiber/PEEK composites reduced λ by 45%, but their high cost and embodied energy limit their adoption in construction [13]. Nanocellulose-modified polyurethane foams exhibit a refined microstructure and a lower λ, yet they still rely on petrochemical-based materials [14]. A recent review highlights the rapid progress in natural fiber composite boards and bio-based adhesives, while also emphasizing the scarcity of panels that are both structurally sound and have low conductivity [15].

At the same time, regulatory and market signals are driving materials toward carbon transparency and circularity. The European Union’s 2024 recast of the Energy Performance of Buildings Directive (EPBD) introduces phased requirements for whole-life carbon assessment and disclosure for new buildings, furthering the demand for low-impact insulation products [16]. Comparative life-cycle assessments (LCAs) indicate that plant-fiber and recycled-fiber materials can achieve lower cradle-to-grave impacts than mineral wool or petrochemical foams, particularly when functional performance is normalized and both biogenic carbon storage and end-of-life are properly accounted for [17]. These findings reinforce the rationale for utilizing recycled textile feedstocks, which not only diverts waste but also reduces processing energy.

From a materials and structural perspective, heat transfer in porous fiber-binder boards is governed by the solid fiber/polymer skeleton, gaseous conduction across the pore spectrum, and a radiative term that increases with cell size [18,19,20,21]. Thus, binder chemistry, volume fraction, and spatial distribution co-tune λ through interfacial contact resistance and the pore-size distribution established during consolidation [22,23,24]. For cellulosic (cotton) fibers, water-based PVAc offers low-toxicity processing and room-temperature film formation. However, moisture absorption can plasticize PVAc and weaken fiber-matrix load transfer on hydroxylated surfaces unless carefully controlled in the formulation [25,26,27]. In contrast, starch binders can be mildly cross-linked to enhance cohesive strength and reduce water uptake [28,29]. PVAc–starch hybrids thus improve wet resistance and interfacial adhesion while maintaining a water-based processing window [30,31].

Despite the growing body of research on natural-fiber insulation materials, there remains limited knowledge on the structure–property relationships of cold-pressed insulation boards produced from heterogeneous post-consumer cotton fibers using fully water-borne hybrid binder systems. In particular, the combined influence of board density and PVAc–starch binder composition on thermal conductivity, mechanical integrity, and moisture-related performance has not been systematically evaluated in such systems.

The objective of this study was therefore to investigate the effect of board density and hybrid PVAc–starch binder content on the thermal, mechanical, and moisture-related behavior of cold-pressed insulation boards manufactured from recycled cotton fibers. Emphasis was placed on understanding how consolidation level and binder formulation influence inter-fiber bonding, pore structure, and the resulting structure–property relationships within a technically relevant density range for building insulation applications.

## 2. Materials and Methods

### 2.1. Recycled Cotton Fibers

**Figure 1:**
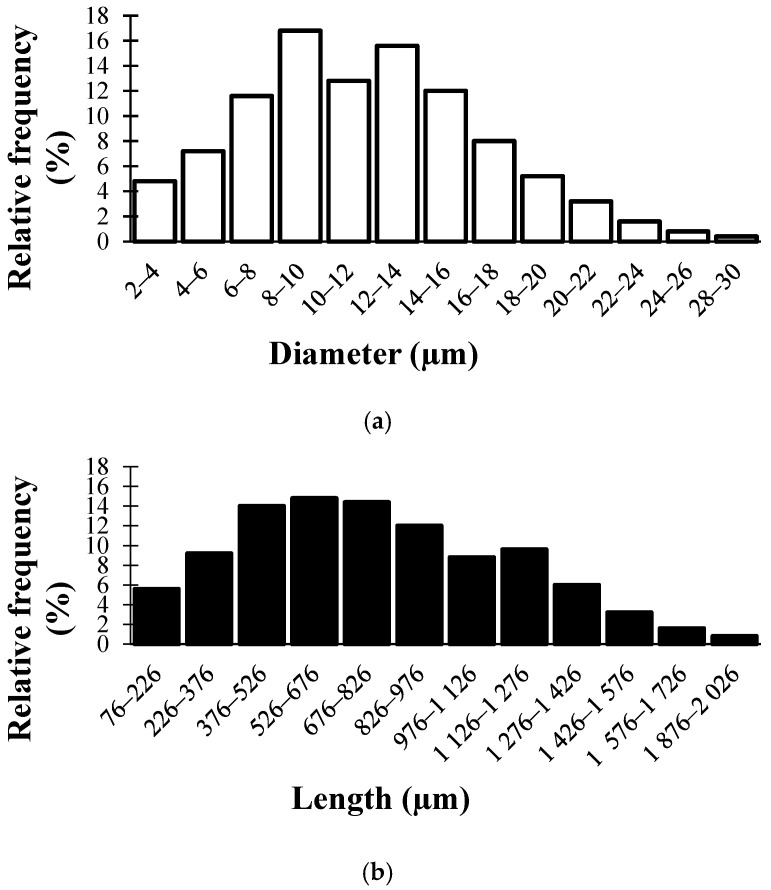
Histograms of fiber diameter (**a**) and fiber length (**b**) for recycled cotton feedstock.

The basic material used in this study was recycled cotton fibers EN-TEX (Enroll CZ, Nová Ves, Czech Republic), originating from mechanically processed post-consumer and post-industrial textile waste. Shredding and fiberizing refer to a purely mechanical size-reduction procedure in which textile residues are progressively torn, cut, and disintegrated into individual fibers, without inducing any chemical modification or thermal exposure. The mechanical shredding and fiberizing were carried out by the material supplier using industrial textile-recycling equipment; no chemical or thermal treatments were applied during this process. To establish the baseline characteristics of the recycled cotton feedstock, the fibers were analyzed in terms of their dimensions, morphology, and chemical composition. The size distribution analysis revealed a broad variability in both diameter and length (Figure 1). Fiber diameters mostly ranged between 8 and 15 µm, while fiber lengths were typically 400–900 µm. This heterogeneity reflects the random breakage of fibers during recycling.

In addition to the recycled fibers, the material contains integrated flame retardants based on aluminum hydroxide (Al(OH)_3_) (ATH) (PENTA s.r.o., Prague, Czech Republic) in weight ration 2–3% weight, applied in the form of a fine powder to the surface of recycled cotton fibers, ensuring classification in reaction-to-fire class E according to the European Technical Assessment ETA-19/0457 [32]. Further additives include antifungal agents compounds Sodium Bicarbonate (NaHCO_3_) (PENTA s.r.o., Prague, Czech Republic) in weight ration 1–2% weight, and Zinc Pyrithione (C_10_H_8_N_2_O_2_S_2_Zn) (PENTA s.r.o., Prague, Czech Republic) in weight ration 1–2% weight, that resist pests and rodents, which are commonly found in commercial cellulose-fiber-based insulation products. Both applied in the form of a fine powder to the surface of recycled cotton fibers; The fibers are supplied without synthetic binders or formaldehyde.

### 2.2. Binder Systems

One of the adhesives used in the composite production was a polyvinyl acetate PVAc-based water dispersion PROFIBOND (Profil Print Technology s.r.o., Všemyslice, Czech Republic). It is a one-component adhesive supplied as a white liquid dispersion with a density of 1.08 ± 0.01 g·cm^−3^, a solids content of 50 ± 1%, a viscosity of 9500 ± 1500 mPa·s, and a pH of 4.75 ± 0.25. The reported density, solids content, viscosity, and pH values correspond to the manufacturer’s technical data sheet. No additional rheological or viscoelastic characterization of the binder was performed within this study. The adhesive provides water resistance corresponding to class D3 and does not contain formaldehyde.

Another adhesive used in the production of the composite boards was corn starch (Herold, Rakovník, Czech Republic), supplied as a fine white powder. The starch used in this study is a polysaccharide composed primarily of amylopectin (≈approximately 72–75%) and amylose (≈approximately 25–28%). Its granules are typically 5–25 µm in diameter, with a bulk density of 0.50 ± 0.05 g·cm^−3^, and a moisture content of less than 13%. The gelatinization temperature is approximately 62–72 °C, at which point the starch swells in water and forms a viscous gel. Corn starch was gelatinized using potable tap water. For each batch, 1.5 L of water was heated to 65–70 °C and the required mass of starch (according to the target formulation) was gradually added under continuous mechanical stirring for 10 min until a homogeneous viscous gel was obtained. The water used for gelatinization served as a processing medium and was not included in the reported composition percentages; the boards were subsequently pressed and dried, and specimens were conditioned to equilibrium moisture content prior to testing.

### 2.3. Microstructural Characterization

Recycled cotton fibers were characterized to establish their baseline morphology and chemistry prior to board fabrication. Their heterogeneous origin from post-consumer and post-industrial waste necessitated an evaluation of potential synthetic residues and inorganic additives, which may impact binder compatibility and composite performance. Morphology was examined using a scanning electron microscope MIRA3 (Tescan Orsay Holding, Brno, Czech Republic) equipped with an energy-dispersive spectroscopy Xplore 15 (Oxford Instruments plc, High Wycombe, UK) system. Images were acquired at 200×, 1000×, 2000×, and 10,000× magnification and analyzed in ImageJ (ImageJ 1.53f, National Institutes of Health, Bethesda, MD, USA), with a total of 250 fibers measured after scale calibration. Elemental composition was determined using the ZAF quantification method. The chemical structure was assessed using FTIR (Nicolet iS20, Waltham, MA, USA; spectra collected in the range of 4000–600 cm^−1^) to identify cellulose functional groups and possible chemical modifications, and Raman spectroscopy WITec alpha 300 (WITec Wissenschaftliche Instrumente und Technologie GmbH, Ulm, Germany) to evaluate cellulose crystallinity and detect synthetic residues.

### 2.4. Preparation of Thermal Insulation Boards

The boards were prepared with target densities of 300–340 kg·m^−3^, the range was set due to a balance between thermal resistance and mechanical integrity in fiber-based materials [33,34,35]. The density of the material depends on the weight fraction of the components cotton fibers, PVAc and cornstarch. Different weight ratios of the three components affect the properties of the composite. After weighing the required proportions of components, the given ratio of PVAc and cornstarch was applied while constantly stirring with a mechanical stirrer LGB (Imalpal S.r.l., San Damaso, Italy) at the constant temperature (20 ± 2 °C) and humidity (65 ± 5%) environment. The reported binder ratios refer to the mass of PVAc aqueous dispersion as supplied (including its water content) and to the dry corn starch powder prior to gelatinization; the additional water used for starch gelatinization was not included in the percentage composition. Test specimens with dimensions of 500 (L) × 300 (W) × 80 (T) mm were produced from the homogenized fiber-binder mixture, placed into molds, and pressed in a universal testing machine TT 2850 (TIRA, GmbH, Schalkau, Germany). The recycled cotton fibers were manually homogenized and distributed into the mold without intentional alignment prior to cold pressing. Therefore, the resulting fiber orientation within the boards can be considered predominantly random within the board plane. The molds were lined with standardized support plates to ensure uniform pressure transfer during the process, and the same support configuration was used for all specimens, regardless of their target density or the specific PVAc-to-starch binder ratio applied in each formulation. The pressing of the mixture of gelatinized starch (65–70 °C) and PVAc adhesive occurred in a cold press, with the entire process completed in a single 2-min cycle. The applied force varied with target density, ranging from 7.5 kN for the lowest-density boards (300 kg·m^−3^) to 25 kN for the highest-density boards (340 kg·m^−3^). The specimens were kept under load for approximately 1 h, then dried in a laboratory drying chamber (Binder FDL, Binder GmbH, Tuttlingen, Germany) at 40 °C for 12 h to remove process water. Subsequently, they were conditioned in a climate chamber (Memmert HPP 750, Memmert GmbH, Schwabach, Germany) at 20 ± 2 °C and 65 ± 5% relative humidity for 14 days to reach equilibrium moisture content.

The complete formulations and pressing parameters are summarized in Table 1.

### 2.5. Bulk Density Determination of Insulation Boards

The bulk density of the insulation boards was determined after conditioning at 20 ± 2 °C and 65 ± 5% relative humidity. Density was calculated as the ratio of the specimen mass to its geometrical volume. The mass of each specimen was measured using a laboratory balance, while the length, width, and thickness were determined using a digital caliper.

The bulk density ρ (kg·m^−3^) was calculated according to Equation (1):

(1) $$\rho =\frac{m}{V}$$

where *m* is the mass of the conditioned specimen (kg) and *V* is the specimen volume (m^3^), calculated from the measured length, width, and thickness.

### 2.6. Thermal Parameters Characterization

Thermal conductivity λ and the thermal diffusivity a were measured using a thermal conductivity device ISOMET 2114 (Applied Precision Ltd., Bratislava, Slovakia) equipped with a needle probe sensor, in accordance with EN ISO 8301 [36]. Measurements were carried out on conditioned specimens under laboratory conditions (20 ± 2 °C, 65 ± 5% relative humidity). Based on the measured values, the volumetric heat capacity ρ·c and thermal diffusivity a were calculated according to Equation (2). For each density level, three independent board specimens were prepared, and thermal conductivity was measured at three different points on each specimen, resulting in nine measurements per variant. The reported values are presented as mean ± standard deviation. The thermal parameters were calculated using the following relationship:

(2) $$a=\frac{\lambda }{\rho \cdot c}$$

where λ is the thermal conductivity (W·m^−1^·K^−1^), c is the specific heat capacity (J·kg^−1^·K^−1^), ρ is the bulk density (kg·m^−3^), ρ·c is the volumetric heat capacity (J·m^−3^·K^−1^), and a is the thermal diffusivity (m^2^·s^−1^).

### 2.7. Moisture-Related Behavior Characterization

Moisture-related behavior of the boards was evaluated in accordance with EN ISO 29767 [37]. Specimens with dimensions of 200 mm (L) × 200 mm (W) × 80 mm (T) were prepared from conditioned boards. Water absorption and thickness swelling were determined by specimens weighed in the conditioned state and reweighed after 24 h of immersion in tap water at a temperature of 20 ± 2 °C. After immersion, excess surface water was removed, and dimensional changes were recorded simultaneously. Measurements were performed under laboratory conditions (20 ± 2 °C, 65 ± 5% relative air humidity). For each density level, five specimens were tested, and the results are reported as mean ± standard deviation. Water absorption (WA) and thickness swelling (TS) were calculated using the following relations:

(3) $$WA(\%)=\frac{m_{2}-m_{1}}{m_{1}}\times 100$$

(4) $$TS(\%)=\frac{t_{2}-t_{1}}{t_{1}}\times 100$$

where $m_{1}$ is the specimen mass after conditioning (g), $m_{2}$ is the specimen mass after immersion (g), $t_{1}$ is the initial specimen thickness (mm), and $t_{2}$ is the thickness after immersion (mm).

### 2.8. Mechanical Properties Characterization

**Figure 2:**
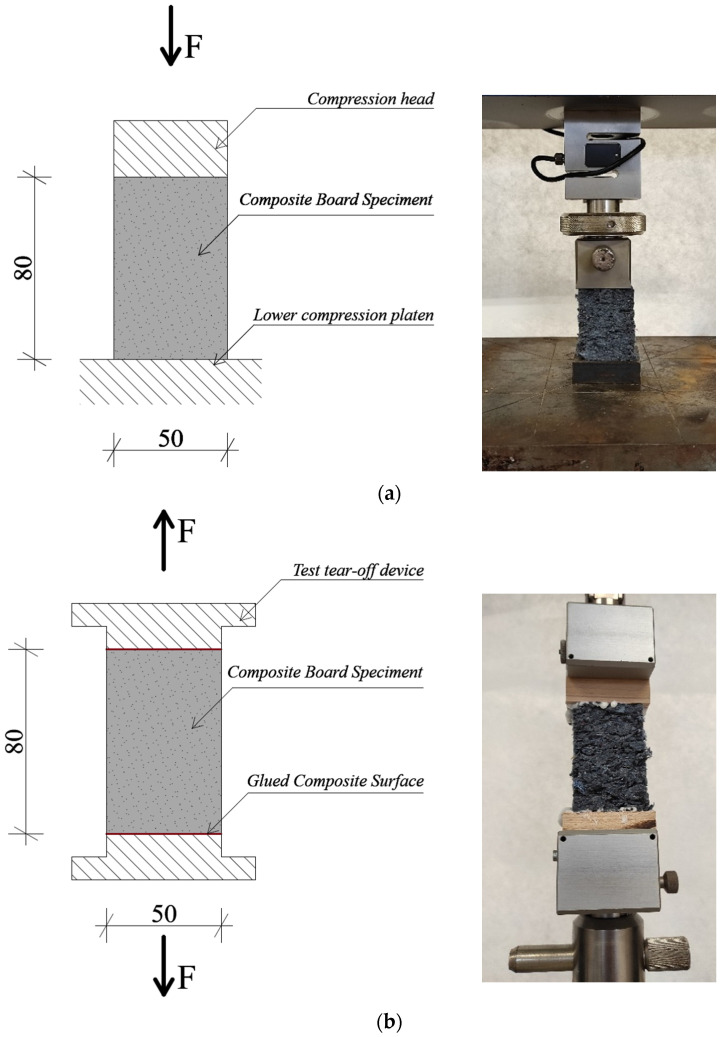
Schematics of the mechanical testing setups: (**a**) compression test configuration according to EN ISO 29469 [38], and (**b**) tensile strength test perpendicular to the board plane according to EN ISO 29766 [39].

Compressive behavior of the thermal insulation boards was determined in accordance with EN ISO 29469 [38] using a universal testing machine TT 2850 (TIRA GmbH, Schalkau, Germany) (Figure 2a). Stress–strain curves were recorded up to 10% relative deformation of the specimen thickness. The load was applied continuously at a crosshead speed corresponding to a strain rate of 10% min^−1^ (≈8 mm·min^−1^ for 80 mm thick samples). For each density level, ten conditioned specimens with dimensions of 50 mm (L) × 50 mm (W) × 80 mm (T) were tested, and the results are reported as mean ± standard deviation. Compressive strength (σ_10_) was calculated as follows:

(5) $$\sigma_{10}=\frac{F}{A}$$

where $\sigma_{10}$ is the compressive stress at 10% deformation (Pa), F is the applied load at 10% deformation (N), and A is the loaded cross-sectional area of the specimen (m^2^).

For the assessment of board cohesion and resistance to tensile loading perpendicular to the board plane, internal bond strength was determined in accordance with EN ISO 29766 [39] using a universal testing machine TT 2850 (TIRA GmbH, Schalkau, Germany) (Figure 2b). Specimens with dimensions of 50 mm (L) × 50 mm (W) × 80 mm (T) were prepared in the conditioned state. The load was applied continuously at a rate of 2 mm·min^−1^ until failure. For each density level, ten specimens were tested, and the results are reported as mean ± standard deviation. Internal bond strength was calculated as follows:

(6) $$\sigma_{ib}=\frac{F}{A}$$

where $\sigma_{ib}$ is the internal bond strength (Pa), F is the maximum load at failure (N), and A is the loaded cross-sectional area of the specimen (m^2^).

The experimental data obtained in this study were processed and evaluated using a combination of graphical and tabular methods. Statistical analyses were performed using STATISTICA 13 (TIBCO Software Inc., Palo Alto, CA, USA), while selected graphs were prepared in Microsoft Excel (Microsoft, Redmond, WA, USA). Basic descriptive statistics were applied to summarize the measured thermal, mechanical, and moisture-related properties of the insulation boards.

Statistical analysis was performed using one-way ANOVA with density as a fixed factor. The level of statistical significance was set at α = 0.05.

## 3. Results and Discussion

### 3.1. Fiber Morphology and Chemical Composition

**Figure 3:**
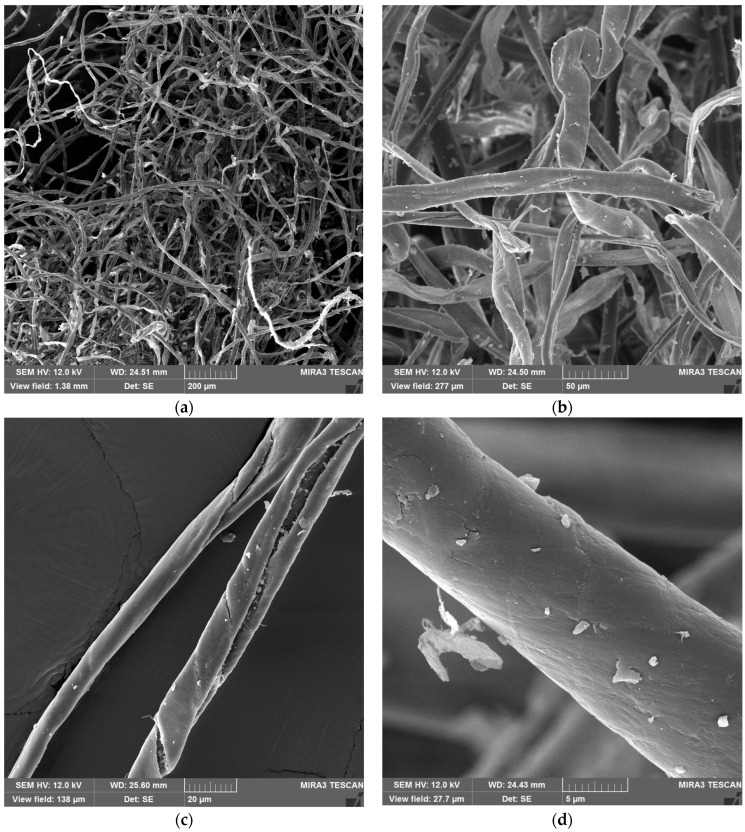
SEM images of recycled cotton fibers at magnifications of (**a**) 200×, (**b**) 1000×, (**c**) 2000×, and (**d**) 10,000×.

SEM analysis confirmed the heterogeneous morphology of recycled cotton fibers. At low magnification, the fibers formed an entangled network with numerous broken ends resulting from mechanical tearing (Figure 3a). Higher magnifications revealed twisted shapes, roughness, and longitudinal cracks (Figure 3b,c), while the highest magnification showed fine cracks and adhering particles (Figure 3d). These features enlarge the surface area for binder adhesion but simultaneously indicate reduced intrinsic fiber strength.

The observed heterogeneity in fiber dimensions reflects the random breakage caused by mechanical recycling and is consistent with previous reports on mechanically recycled cotton [40]. This variability in aspect ratio plays a dual role in composite performance. Fibers with higher aspect ratios can act as reinforcing elements that bridge across the binder matrix, improving tensile properties [41,42]. In contrast, shorter and thicker fibers increase the available interfacial area for binder adhesion, thereby enhancing cohesion but contributing less to load transfer [43]. Moreover, aspect ratio not only governs mechanical integrity but also affects the porosity and thermal insulation performance of fiber networks, as shown in simulation studies [44].

FTIR analysis confirmed cellulose as the dominant component, with characteristic O-H and C-H absorption bands, while some spectra also showed matches to blended textiles such as cotton–viscose (≈86%) and cotton–elastane (≈84%). These results demonstrate that the fibers originated from post-consumer waste rather than pure cotton (Table 2). Raman spectroscopy complemented these findings by identifying additional constituents, including polyester (≈69%), polypropylene (≈96%), and magnesium sulfate heptahydrate (≈66%). The detection of synthetic polymers and inorganic additives highlights the industrial processing history of the fibers and supports the conclusion that the recycled feedstock represents a complex mixture of natural, synthetic, and inorganic components (Table 2).

Some spectra, however, indicated blended textiles, such as cotton–viscose or cotton–elastane, consistent with recent studies on mixed natural fibers [45,46]. Raman spectroscopy complemented these findings by identifying polyester, polypropylene, and magnesium sulfate heptahydrate, also reported in analyses of recycled cotton textiles [45]. The presence of hydroxyl-rich cellulose domains may enhance bonding with the PVAc–starch binder, whereas synthetic polymers provide less chemical interaction and may act as weak points. The detection of magnesium sulfate reflects the industrial finishing of cotton, supporting the conclusion that the fibers represent a heterogeneous mixture of natural, synthetic, and inorganic components typical of post-consumer waste.

To complement the spectroscopic analyses, EDS was performed to verify the elemental composition of the recycled fibers and to detect possible inorganic residues. The measurements confirmed carbon and oxygen as the dominant elements (≈34–68 wt.% C; 31–48 wt.% O), consistent with cellulose as the main component. Minor amounts of aluminum, sodium, and boron, as well as traces of rubidium, were also detected (Table 3). These findings support the FTIR and Raman results, indicating that the recycled fibers are chemically heterogeneous and contain residues of textile processing additives.

Such inorganic residues may slightly improve binder adhesion through increased surface activity but also contribute to chemical heterogeneity, potentially introducing weak points in the composite. Similar observations on residual elements affecting the interfacial properties of recycled fibers have been reported in recent studies [46,47].

### 3.2. Composite Thermal Insulation Board Morphology

**Figure 4:**
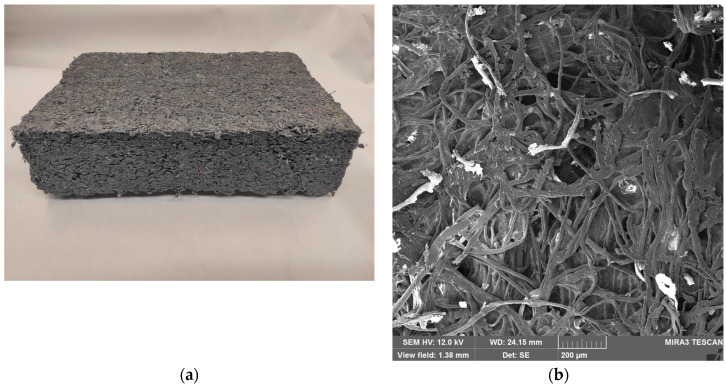
Morphology of recycled cotton insulation boards: (**a**) macroscopic view and (**b**) SEM image at magnifications of 200×.

The morphology of the composite boards is shown in Figure 4. At the macroscopic scale, the boards exhibited a rough surface with visible fiber fragments and inclusions, reflecting the heterogeneous nature of the recycled textile feedstock (Figure 4a). SEM analysis confirmed a porous network of entangled fibers interconnected by PVAc–starch binder domains (Figure 4b). The binder was observed in both continuous film and discrete granular forms, indicating its dual role in fiber bridging and pore filling.

Such heterogeneity, typical of natural-fiber composites, is linked to strength variability [48,49], while the high porosity supports low thermal conductivity, as reported in porous fiber-based materials. At the same time, the open and interconnected pore structure may facilitate moisture uptake through capillary transport and sorption on hydrophilic fiber surface [50,51]. Excessive voids, however, may act as stress concentrators and limit structural reliability.

### 3.3. Bulk Density of Insulation Boards

For each formulation variant, multiple insulation boards were produced, and their bulk density was determined after conditioning. The measured bulk density values showed low variability within each density variant, with deviations not exceeding ± 5%.

The density values reported in tables and figures (300, 310, 320, 330, and 340 kg·m^−3^) represent nominal target density classes used for sample designation and comparison. All reported material properties correspond to specimens whose measured bulk density fell within the defined tolerance range. In the present study, density and binder content were intentionally varied together to reflect realistic technological adjustment during board consolidation. While bulk density directly governs structural compactness and thermal transport behavior, the gradual increase in PVAc content ensured sufficient inter-fiber bonding at higher compaction levels. Therefore, the observed property trends represent the integrated effect of densification and binder adaptation rather than the isolated influence of a single variable.

### 3.4. Thermal Parameters of the Thermal Insulation Board

Table 4 presents the mean values of thermal parameters (± standard deviation) obtained from nine measurements per density variant. Thermal conductivity showed a slight decreasing trend with higher density. In contrast, specific heat capacity varied across the tested range, reflecting differences in the heat storage capacity of the composites. Thermal diffusivity exhibited a non-monotonic dependence on density, resulting from the combined influence of thermal conductivity and volumetric heat capacity.

In the present study, the thermal conductivity of recycled cotton fiber insulation boards ranged from 0.0710 to 0.0739 W·m^−1^·K^−1^ at bulk densities of 300–340 kg·m^−3^, which corresponds well with values reported for bio-based and recycled insulation materials. Heat transfer in porous fiber boards is governed by the combined contributions of solid conduction through the fiber–binder skeleton, gaseous conduction through the pore network, and radiative heat transfer within the pores, as has been demonstrated in recent studies on natural and fiber-derived insulation materials [52,53]. Increasing bulk density typically reduces pore size and limits gaseous and radiative pathways while increasing the connectivity of the solid phase [53]. A balance between suppressed gas/radiative contributions and enhanced solid conduction can therefore result in a shallow minimum in thermal conductivity at intermediate densities, which is consistent with theoretical frameworks that concurrently assess the contributions of multiple heat transfer modes in porous structures [54]. Comparable thermal conductivity values of 0.050–0.065 W·m^−1^·K^−1^ were reported in recent study for natural fiber-reinforced insulation composites, emphasizing the influence of fiber structure and porosity on heat transfer [55]. Another study demonstrated that recycled textile-based insulation materials may reach *λ* values up to 0.140 W·m^−1^·K^−1^, depending on density and processing conditions, indicating that the results obtained in this study fall within the broader performance range of recycled fiber insulation systems [56]. In addition to thermal conductivity, the studied insulation boards exhibited relatively high specific heat capacity, which is characteristic of natural fiber-based materials and contributes to enhanced thermal inertia and delayed heat transfer through building envelopes [55,57].

One of the investigated variants exhibited noticeably deviating thermal properties compared to the trend of the remaining samples, which can be attributed to the inherent heterogeneity of recycled textile-based composites. The slight increase in λ at the highest density may indicate that solid-phase heat transfer becomes more dominant due to increased inter-fiber contact and a higher degree of binder continuity, as increased interfacial contact area and interphase continuity have been shown to enhance effective thermal conductivity in fibrous composites [58]. In addition, local heterogeneity (e.g., binder-rich regions or locally compacted zones) can partially fill pores and create preferential heat-transfer pathways, since higher density and reduced porosity in recycled textile-based composites have been experimentally associated with increased λ due to more extensive solid conduction networks [59]. This is consistent with broader findings that microstructural arrangement, phase distribution, and the formation of continuous conductive pathways strongly govern heat transport in heterogeneous polymer and fiber composites [60]. The insulation boards were produced predominantly from recycled cotton fibers, while the binder system consisted of a PVA–starch mixture. In addition, fractions of synthetic fibers, originating from recycled textile waste, were present in the material, as confirmed by previous material analyses. Recent studies have demonstrated that even limited amounts of synthetic fibers, such as polyester or elastane, can locally affect heat transfer pathways due to their different intrinsic thermal properties compared to cotton fibers [61,62]. Furthermore, uneven distribution of polymer-based binders may partially fill pore spaces and increase fiber–fiber contact, leading to locally enhanced conductive heat transfer within fibrous insulation materials [63,64]. The observed deviation is most likely related to local material heterogeneity inherent in recycled textile composites.

### 3.5. Moisture-Related Behavior of the Thermal Insulation Board

Table 5 presents the mean values (±standard deviation) of water absorption (WA) and thickness swelling (TS) obtained from five specimens per density variant. Both parameters exhibited a clear decreasing trend with increasing board density, indicating lower moisture uptake and dimensional changes in denser composites. The observed variations among samples are attributed to the heterogeneous structure of the recycled fibers and the non-uniform distribution of the PVAc–starch binder.

Water absorption and thickness swelling decreased with increasing board density, confirming that reduced porosity and higher binder content and improved binder distribution limit moisture penetration. In addition, denser boards contained a proportionally higher amount of the PVAc–starch adhesive, which filled voids more effectively and formed a partially continuous phase around fibers, thereby limiting capillary water uptake. Similar relationships between density and moisture uptake have been reported for natural-fiber composites, where micro voids and hydrophilic cellulose regions dominate water diffusion [49,50]. The obtained absorption levels (≈18–22%) are slightly higher than those reported for jute or flax composites, reflecting the inherently porous and hydrophilic nature of recycled cotton fibers. However, previous studies indicate that the application of hydrophobic surface treatments can effectively mitigate this effect and enhance dimensional stability under humid conditions [51].

### 3.6. Mechanical Properties of the Thermal Insulation Board

**Figure 5:**
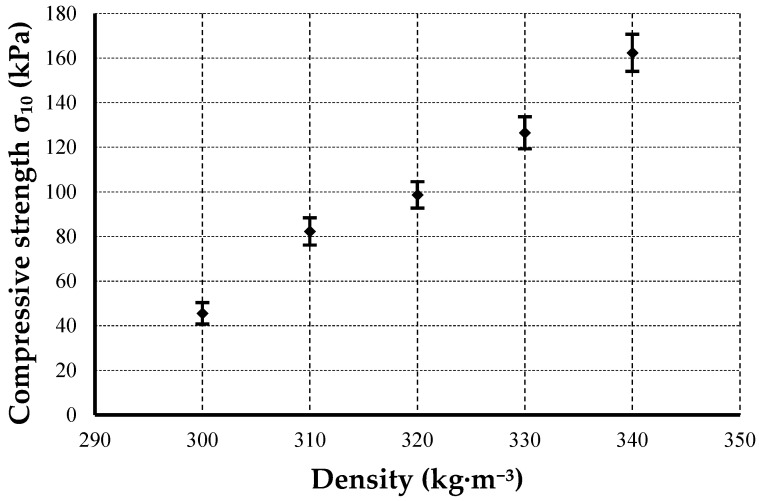
Variation in compressive strength (σ_10_) with density for thermal insulation boards at 10% deformation (EN ISO 29469 [38], *n* = 10). Vertical error bars represent standard deviations.

Figure 5 and Table 6 present the mean compressive strength values (±standard deviation) (σ_10_) determined at 10% relative deformation for ten specimens per density variant, tested according to EN ISO 29469. The results show a consistent increase in compressive strength with increasing board density and with higher adhesive content, ranging from approximately 46 to 162 kPa.

Compressive strength increased nearly linearly with density and higher adhesive content. One-way analysis of variance (ANOVA) was performed to evaluate the effect of density on compressive strength σ_10_. The results confirmed a statistically significant effect of density (F(4,45) = 451.18, *p* < 0.001) at a significance level of α = 0.05). This behavior can be explained by microstructural densification of the fiber network. Increasing density promotes more frequent fiber-fiber contacts and a more continuous binder phase, which enhances stress transfer within the composite. At the same time, reduced void volume limits local instability and stress concentrations, contributing to the nearly linear increase in compressive strength with density. The improved binder fraction at greater compaction levels also contributed to stronger inter-fiber adhesion and more efficient load distribution. This trend aligns with findings reported for bio-based insulation and starch-reinforced composites, where densification reduces void content and enhances interfacial adhesion between fibers and the matrix [65,66]. The obtained strength levels (≈46–162 kPa) are consistent with previously published data for recycled or natural fiber-based panels [67]. The relatively low variation across samples demonstrates good fiber distribution and uniform binder dispersion. Similar improvements in compressive behavior due to higher starch or polymeric binder content were also observed in natural fiber-reinforced starch bio-composites [68,69].

**Figure 6:**
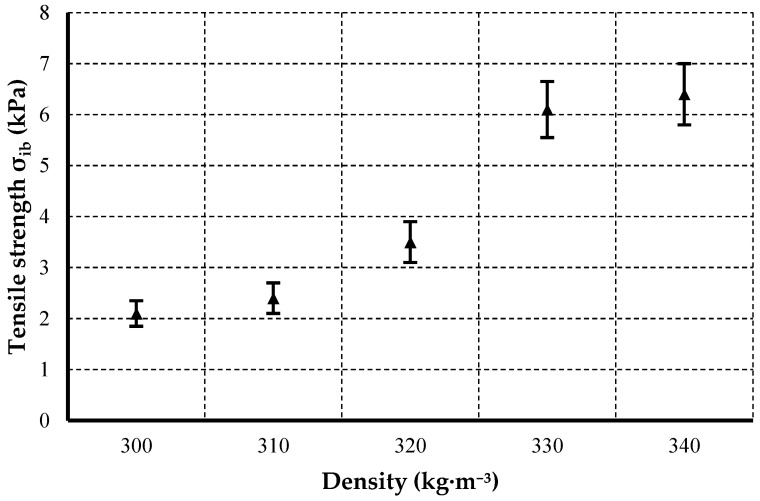
Variation in internal bond strength (σ_ib_) with density for thermal insulation boards (EN ISO 29766 [39], *n* = 10). Vertical error bars represent standard deviations.

Figure 6 and Table 7 show the internal bond strength (±standard deviation) of thermal insulation boards, as determined according to EN ISO 29766 [39]. The mean tensile strength increased with board density from approximately 2 to 6 kPa.

The internal bond strength increased from ~2.1 to ~6.4 kPa with increasing density and adhesive content. One-way analysis of variance (ANOVA) confirmed a statistically significant effect of density on internal bond strength σ_ib_ (F(4,45) = 210.61, *p* < 0.001; α = 0.05). The increase in internal bond strength with density can be attributed to improved interfacial cohesion within the fiber-binder network. Higher compaction increases the effective bonded area between fibers and promotes a more continuous adhesive phase, which enhances tensile stress transfer perpendicular to the board plane. Simultaneously, the reduction in internal voids decreases the likelihood of interlayer delamination and premature cohesive failure. This relationship is in line with observations in wood-fiber insulation boards, where increased adhesive content or compaction boosted tensile strength perpendicular to the surface by 20–36% [70]. The σ_ib_ values obtained here (≈2–6 kPa) are comparable to internal bond strengths reported in fiberboard panels made from recycled or natural fibers using bio-based binders [71]. Similar densification effects where increasing density or binder content enhances tensile integrity have been documented in other fiber–polymer systems, reflecting improved interfacial bonding and reduced void volume [72].

## 4. Conclusions

This study demonstrated that recycled cotton fibers can be effectively transformed into cohesive fiber-based insulation boards using a fully water-borne PVAc–starch binder system and a low-temperature cold-pressing process. The resulting materials exhibited densities between 300 and 340 kg·m^−3^ and showed a balanced combination of thermal and mechanical performance, achieving thermal conductivity values of 0.0710–0.0739 W·m^−1^·K^−1^, compressive strengths of up to 160 kPa, and internal bond strengths exceeding 6 kPa. Increasing board density and binder content enhanced inter-fiber stress transfer and cohesion while preserving the lightweight and recyclable character of the fibrous structure. Although the materials remained moderately hydrophilic, improved binder distribution and reduced open porosity effectively mitigated moisture uptake. The demonstrated fabrication route, which is fully water-borne, formaldehyde-free, and based on room-temperature cold pressing followed by low-temperature drying, provides a potentially low-energy and scalable pathway, although the overall process energy demand depends on drying efficiency and scale-up conditions for the valorization of post-consumer cotton fibers into functional technical textile materials for insulation and building-related applications. Overall, the findings confirm that recycled cotton fiber-based boards represent a promising class of circular, low-impact materials combining satisfactory mechanical integrity with favorable thermal performance. Future work should focus on process optimization, moisture resistance enhancement, and scale-up considerations to support consistent industrial production.

## Figures and Tables

**Table 1:** Composition of Test Specimens with Different Densities and Adhesives Ratio.

Density (kg·m^−3^)	Cotton Fibers (%) *	PVAc (%) *	Corn Starch (%) *	Pressing Force (kN)
300	80	10	10	7.5
310	78	12	10	12
320	75	15	10	16
330	72	18	10	20
340	70	20	10	25

* Weight percent based on weighed component mass (PVAc aqueous dispersion and dry corn starch).

**Table 2:** Identified components of recycled cotton fibers by FTIR and Raman spectroscopy.

Method	Identified Components	Notes/Match (%)
**FTIR**	Cellulose	Main component
	Cotton–viscose blend	86% match
	Cotton–elastane blend	84% match
**Raman**	Polyester	69% match
	Polypropylene	96% match
	Magnesium sulfate heptahydrate	66% match

**Table 3:** Elemental composition (Mass%) of recycled cotton fiber samples measured by EDS.

Element	Sample 1	Sample 2	Sample 3
Boron (B)	2.96 ± 0.45	0.84 ± 0.26	–
Carbon (C)	33.77 ± 2.53	68.15 ± 3.94	46.71 ± 3.07
Oxygen (O)	43.19 ± 6.29	31.01 ± 6.76	47.79 ± 7.34
Sodium (Na)	5.08 ± 4.07	–	–
Aluminum (Al)	10.84 ± 9.34	–	5.50 ± 10.52
Rubidium (Rb)	4.17 ± 58.36	–	–

**Table 4:** Thermal Properties (mean values ± standard deviation) of Recycled Cotton Fiber Thermal Insulation Boards.

Density (kg·m^−3^)	Thermal Conductivity (W·m^−1^·K^−1^)	Specific Heat Capacity (J·kg^−1^·K^−1^)	Thermal Diffusivity (×10^−6^ m^2^·s^−1^)
300	0.0738 ± 0.0003	1587 ± 93	0.155 ± 0.013
310	0.0730 ± 0.0006	1419 ± 59	0.166 ± 0.010
320	0.0724 ± 0.0003	1278 ± 85	0.177 ± 0.010
330	0.0710 ± 0.0006	916 ± 116	0.235 ± 0.044
340	0.0739 ± 0.0006	1294 ± 94	0.168 ± 0.011

**Table 5:** Short-term water absorption and dimensional stability of Recycled Cotton Fiber Thermal Insulation Boards.

Density (kg·m^−3^)	Height Change (mm)	Depth Change (mm)	Width Change (mm)	Weight Gain (g)	Water Absorption WA (%)	Thickness Swelling TS (%)
300	+1.8 ± 0.3	+4.4 ± 0.5	+1.6 ± 0.4	+258 ± 10	22.1 ± 0.9	5.6 ± 0.4
310	+1.5 ± 0.2	+4.3 ± 0.6	+1.3 ± 0.5	+240 ± 11	21.7 ± 1.0	5.1 ± 0.5
320	+1.2 ± 0.3	+3.9 ± 0.5	+1.1 ± 0.4	+230 ± 12	20.5 ± 0.8	4.8 ± 0.6
330	+1.0 ± 0.2	+3.5 ± 0.4	+1.0 ± 0.3	+220 ± 9	19.0 ± 0.7	4.3 ± 0.4
340	+0.9 ± 0.2	+3.2 ± 0.3	+0.8 ± 0.2	+210 ± 8	18.2 ± 0.6	3.9 ± 0.5

**Table 6:** Compressive strength (mean values ± standard deviation) (σ_10_) of thermal insulation boards at 10% deformation (EN ISO 29469, *n* = 10).

Density (kg·m^−3^)	Compressive Strength σ_10_ (kPa)	Coefficient of Variation (%)
300	45.6 ± 4.8	10.5
310	82.3 ± 6.1	7.4
320	98.7 ± 5.9	6.0
330	126.5 ± 7.2	5.7
340	162.4 ± 8.3	5.1

**Table 7:** Internal bond strength (mean values ± standard deviation) of thermal insulation boards at different densities determined according to EN ISO 29766 [39] (*n* = 10).

Density (kg·m^−3^)	Tensile Strength σ_ib_ (kPa)	Coefficient of Variation (%)
300	2.10 ± 0.25	11.9
310	2.40 ± 0.30	12.5
320	3.50 ± 0.40	11.4
330	6.10 ± 0.55	9.0
340	6.40 ± 0.60	9.4

## Data Availability

The original contributions presented in this study are included in the article. Further inquiries can be directed to the corresponding author.

## Abbreviations

*A*	Loaded cross-sectional area of specimen (mm^2^)
*a*	Thermal diffusivity (m^2^·s^−1^)
*c*	Specific heat capacity (J·kg^−1^·K^−1^)
*d*	Specimen thickness (m)
*F*	Load (N)
*λ*	Thermal conductivity (W·m^−1^·K^−1^)
L	Specimen length (mm)
*m*_0_	Mass of specimen before immersion (g)
*m*_1_	Mass of specimen after immersion (g)
*ρ*	Density (kg·m^−3^)
*ρ* · *c*	the volumetric heat capacity (J·m^−3^·K^−1^)
*σ*_10_	Compressive strength at 10% deformation (kPa)
*σ_ib_*	Internal bond strength (tensile strength perpendicular to board plane) (kPa)
*t*_0_	Initial specimen thickness before immersion (mm)
*t*_1_	Specimen thickness after immersion (mm)
T	Specimen thickness (mm)
*TS*	Thickness swelling (dimensional change after immersion) (%)
*V*	Specimen volume (m^3^)
W	Specimen width (mm)
*WA*	Water absorption (mass increase after immersion) (%)
